# Long-term impacts of complete revascularization on clinical outcomes in patients with coronary chronic total occlusion

**DOI:** 10.1016/j.heliyon.2024.e40326

**Published:** 2024-11-12

**Authors:** Tae Oh Kim, SeHee Kim, Min-Ju Kim, Do-Yoon Kang, Pil Hyung Lee, Soo-Jin Kang, Cheol Whan Lee, Young-Hak Kim, Jong-Young Lee, Seung-Whan Lee

**Affiliations:** a Division of Cardiology, Department of Internal Medicine, Asan Medical Center, University of Ulsan College of Medicine, Seoul, Republic of Korea; b Division of Biostatistics, Department of Clinical Epidemiology and Biostatistics, Asan Medical Center, University of Ulsan College of Medicine, Seoul, Republic of Korea; c Division of Cardiology, Department of Internal Medicine, Kangbuk Samsung Hospital, Sungkyunkwan University School of Medicine, Seoul, Republic of Korea

**Keywords:** Coronary artery disease, Chronic total occlusion, Complete revascularization, Percutaneous coronary intervention, Optimal medical treatment

## Abstract

The impact of complete revascularization (CR), achieved through the recanalization of coronary chronic total occlusions (CTOs), on long-term patient outcomes remains uncertain.

To evaluate this in patients who achieved CR after CTO-PCI with those who did not due to deferred CTO-PCI, the Asan Medical Center Registry was reviewed to identify coronary artery disease (CAD) patients with CTOs treated between January 2003 and December 2018. Patients were included with single-vessel disease with CTO and with multivessel disease who had undergone revascularization for non-CTO lesions. These subjects were divided into those who achieved CR with CTO-PCI and those who did not due to deferred CTO-PCI. Their outcomes were compared following 1:1 propensity score matching. Of the 2746 enrolled CAD patients with CTOs, 1837 achieved CR with CTO-PCI and 909 did not. Propensity score matching yielded 653 patient pairs. The CR-achieving group had a significantly lower 10-year risk of the primary composite outcome of death, myocardial infarction, stroke, or repeat revascularization (hazard ratio [HR]: 0.57; 95 % confidence interval [CI]: 0.46–0.72; P < 0.001), as well as significantly lower risks of death (HR: 0.66; 95 % CI: 0.51–0.87; P = 0.003) and repeat revascularization (HR: 0.67; 95 % CI: 0.48–0.95; P = 0.023). CR was beneficial in all subgroups, including patients with major cardiovascular risk factors such as older age, hypertension, diabetes, and advanced CAD. Compared with incomplete revascularization, CR may significantly reduce the 10-year incidence of major adverse cardiac events in patients with CTO.

## Teaser

Condensed Abstract: Analysis of a real-world registry of patients with coronary chronic total occlusion (CTO) revealed that treatment decisions were influenced by revascularization risk, favoring aggressive interventions in healthier patients and conservative interventions for less healthy patients. To determine whether the benefits of achieving complete revascularization (CR) with CTO-percutaneous intervention (CTO-PCI) were overestimated, between-group differences were adjusted by propensity score matching. CR with CTO-PCI significantly reduced the risks of death, myocardial infarction, stroke, or repeat revascularization compared with incomplete revascularization with deferred CTO-PCI. These benefits included significantly lower mortality and repeat revascularization rates, underscoring the clinical advantages of achieving CR in CTO patients.

## Abbreviations and acronyms

CABG: coronary artery bypass grafting
CAD =: coronary artery disease
CI =: confidence interval
CR =: complete revascularization
CTO =: chronic total occlusion
DES =: drug-eluting stent
HR =: hazard ratio
MI =: myocardial infarction
OMT =: optimal medical treatment
PCI =: percutaneous coronary intervention
RCT =: randomized controlled trial
STEMI =: ST-elevation myocardial infarction
TIMI =: thrombolysis in myocardial infarction

## 1 Introduction

Chronic total occlusions (CTOs) are conditions in which the coronary arteries are completely blocked by atherosclerosis, limiting adequate blood flow to the myocardium. This can lead to reduced cardiac function and the progression of complex cardiovascular disease [1]. Despite their clinical significance, the length of the lesions and their complicated pathology make revascularization a challenge. Recent advances in coronary intervention have improved the success rates of percutaneous coronary interventions (PCIs) targeting CTOs (known as CTO-PCI) and reduced the risks of complications. However, only about 10 % of patients with CTOs undergo these procedures [2]. The widespread reluctance to perform CTO-PCIs is due to uncertainties about their effectiveness, as well as technical difficulties [3,4]. Observational studies and randomized controlled trials (RCTs) have not yet shown that CTO-PCI has clear long-term benefits when compared with optimal medical therapy (OMT) for the treatment of CTO, leading to a cautious approach to aggressive revascularization strategies [5,6].

Evaluations of the long-term benefits of CTO-PCI must also include the potential complications of unsuccessful procedures, as well as the impact of untreated non-CTO lesions within these patient populations, as these factors could reduce the long-term benefits of successful CTO-PCI. Hence, comparing long-term clinical outcomes in patients who achieve complete revascularization (CR) or not, particularly those with successfully treated non-CTO lesions or single-vessel CTO, may provide insight into the true effectiveness of CTO-PCI. This retrospective study has therefore utilized data from an open-label, real-world clinical practice registry to comprehensively evaluate the effectiveness of CTO-PCI in various settings. The goal of this study was to evaluate the impact of CR resulting from CTO recanalization on long-term patient outcomes.

## 2 Methods

### 2.1 Study population and data sources

The registry-based analysis conducted in this study evaluated consecutive patients with coronary CTO who underwent either revascularization or not between January 1, 2003 and December 30, 2018, at Asan Medical Center (Seoul, Republic of Korea). This registry was designed to evaluate the real-world outcomes in patients with CTO (i.e., chronic total occlusion of the three major epicardial vessels with a reference vessel diameter >2.5 mm) who underwent either CTO revascularization or not. To assess the impact of CR by PCI on long-term outcomes in coronary artery disease (CAD) patients with CTO, patients who underwent CABG and those with multivessel CAD who did not receive PCI for significant non-CTO lesions were excluded from the analysis. To focus on the efficacy of successful CTO-PCI, patient who underwent failed CTO-PCI were also excluded. The registry contains information on patient demographics, cardiovascular risk factors, clinical presentation, hemodynamic status, left ventricular function, extent of disease, procedural details, and in-hospital and follow-up outcomes. All data were recorded by independent research personnel in dedicated PCI databases. The study protocol was approved by the Institutional Review Board of Asan Medical Center (IRB approval number: 2023–0982). Informed patient consent was waived due to the retrospective nature of the study.

### 2.2 Study procedures

The choice of treatment strategy for CTO was at the discretion of the treating physician and/or the patient, based on various clinical and anatomic factors determined by diagnostic coronary angiography. Patients with more than single-vessel CTO disease were included, thus also allowing for cases with revascularization of non-CTO lesions. Standard PCI techniques were conducted in the study cases, with selection of the type of drug-eluting stent (DES) not based on specific guidelines for clinical or anatomic conditions. All of the included patient subjects who underwent PCI received a loading dose of aspirin and a P2Y12 receptor inhibitor (clopidogrel, prasugrel, or ticagrelor) before or during the procedure. Following the procedure, the patients received dual antiplatelet therapy for a minimum of 12 months, followed by either aspirin or a P2Y12 receptor inhibitor for an indefinite time, regardless of the type of DES used. Patients were also prescribed secondary cardiovascular disease prevention medications in accordance with current guidelines [7,8].

### 2.3 Follow-up and endpoints

Patients were followed-up by reviewing hospital records, outpatient visit notes, and all other pertinent medical records. To ensure the validity of complete follow-up data on mortality, the vital status and date of death for each of the relevant study subjects were obtained from their electronic medical records and cross-checked with the National Health Insurance Service of South Korea. Vital status information was completed for all included patients. The primary outcome of this study was the composite of all-cause mortality, spontaneous myocardial infarction (MI), stroke, or repeat revascularization. Secondary outcomes included each component of the primary composite outcome, as well as cardiac death and CTO-related repeat revascularization. All outcomes were adjudicated using standard endpoint definitions [9,10]. Survival was evaluated by determining the incidence of all-cause mortality as this parameter is impartial and is commonly used to report deaths in clinical trials and observational studies. Spontaneous MI was defined as the occurrence of new-onset ischemic symptoms or signs with cardiac enzyme elevation above the upper limit of normal, requiring rehospitalization (i.e., emergency admission with a primary diagnosis of MI). Periprocedural MI was not included because of inconsistent definitions and controversial prognostic implications [11,12]. Stroke was defined as the sudden onset of neurological symptoms (e.g., dizziness, numbness, aphasia, and dysarthria) resulting from cerebral vascular lesions (e.g., hemorrhage, embolism, thrombosis, and ruptured aneurysm) lasting >24 h. Repeat revascularization included any subsequent PCI or surgical bypass of a treated or untreated vessel, regardless of whether the procedure was clinical or ischemia-driven. Specifically, CTO-related repeat revascularization was defined as revascularization of the same lesion based on a patient's clinical course after establishment of the initial treatment strategy for CTO.

### 2.4 Study definitions

A CTO was characterized as a complete occlusion with a TIMI flow grade of 0 antegrade through the affected segment for >3 months, as determined by the operator based on clinical and angiographic features and/or prior imaging. Successful CTO-PCI was defined as the restoration of the TIMI flow to grade 3 with residual stenosis <30 %, as determined by the operator. Failure of CTO-PCI was defined as a failure to cross the occlusion or reduce the occlusion to <50 % in the target CTO. Complete revascularization was defined as successful PCI of the target lesions and a postprocedural stenosis of <50 % in all major epicardial coronary arteries within a vessel diameter ≥2.5 mm, as visually estimated [13].

### 2.5 Statistical analysis

Long-term outcomes following initial CTO treatment were analyzed in both the overall and 1:1 propensity score-matched cohorts. For baseline characteristics, normally distributed continuous variables were compared using Student's *t*-tests, whereas non-normally distributed continuous variables were compared with Wilcoxon rank-sum tests. Categorical variables were compared using the χ^2^ test or Fisher's exact test, as appropriate. 1:1 propensity score matching was employed to rigorously control for baseline characteristics across the two groups. Propensity score-matched pairs were determined using a greedy algorithm with a caliper of 0.2 standard deviations of the logit of the propensity score (c-statistic = 0.821; Hosmer–Lemeshow test P = 0.388 supporting goodness of fit). The adequacy of the propensity score matching method was considered satisfactory if overall balance was achieved, as indicated by a standardized mean difference of <10 %.

The cumulative incidence of outcomes was calculated using the Kaplan–Meier method and compared using log-rank tests. In matched cohorts, Cox regression with robust standard errors was used. Subgroup analyses focused on clinically relevant variables, including age, sex, hypertension, diabetes, heart failure, previous MI, chronic kidney disease, CTO location, ejection fraction, presentation of acute coronary syndrome, and extent of disease. Interaction tests were performed to assess the heterogeneity of treatment effects across these subgroups.

Independent predictors of primary and secondary clinical outcomes were identified by multivariate Cox regression analyses. Among the baseline clinical and anatomical covariates listed in Table 1, those having P-values <0.20 in univariate analyses were included in the multivariate Cox proportional hazards models. The determination of multivariate models was determined through backward elimination methods (retention threshold: P < 0.05). The proportional hazards assumption for all variables was confirmed by the Schoenfeld residuals test, revealing no significant violations of the assumption.

**Table 1:** Baseline study patient characteristics according to the initial treatment strategies for chronic total occlusion.

Variables	Unadjusted				Propensity score-matched		
	CR (n = 1837)	Non-CR (n = 909)	P-Value	Standardized Difference (%)	CR (n = 653)	Non-CR (n = 653)	Standardized Difference(%)
Year of procedure							
2003–2007	487 (26.5 %)	31 (3.4 %)	<0.001	59.9 %	68 (10.4 %)	30 (4.6 %)	3.7 %
2008–2018	1350 (73.5 %)	878 (96.6 %)			585 (89.6 %)	623 (95.4 %)	
Age (years)	61.1 ± 10.6	66.3 ± 10.6	<0.001	49.0 %	64.8 ± 9.8	64.8 ± 10.9	0.8 %
Male	1508 (82.1 %)	733 (80.6 %)	0.36	3.7 %	525 (80.4 %)	538 (82.4 %)	5.1 %
Body mass index (kg/m^2^)	25.5 ± 3.2	24.8 ± 3.4	0.01	21.5 %	25.0 ± 3.1	25.1 ± 3.3	3.5 %
Diabetes mellitus							
Any	558 (30.4 %)	382 (42.0 %)	<0.001	24.4 %	240 (36.8 %)	250 (38.3 %)	3.2 %
Requiring insulin	88 (4.8 %)	64 (7.0 %)	0.02	9.5 %	45 (6.9 %)	42 (6.4 %)	1.8 %
Hypertension	1091 (59.4 %)	630 (69.3 %)	<0.001	20.8 %	425 (65.1 %)	434 (66.5 %)	2.9 %
Hyperlipidemia	1385 (75.4 %)	870 (37.3 %)	<0.001	24.1 %	556 (85.1 %)	542 (83.0 %)	5.9 %
Current smoker	512 (27.9 %)	244 (26.8 %)	0.57	2.3 %	167 (25.6 %)	179 (27.4 %)	4.2 %
Previous MI	180 (9.8 %)	137 (15.1 %)	<0.001	16.0 %	83 (12.7 %)	79 (12.1 %)	1.9 %
Previous PCI	453 (24.7 %)	273 (30.0 %)	0.01	12.1 %	191 (29.2 %)	186 (28.5 %)	1.7 %
Previous stroke	123 (6.7 %)	107 (11.8)	<0.001	17.6 %	63 (9.6 %)	61 (9.3 %)	1.0 %
Previous heart failure	52 (2.8 %)	55 (6.1 %)	<0.001	15.7 %	34 (5.2 %)	35 (5.4 %)	7.0 %
Peripheral artery disease	52 (2.8)	51 (5.6)	<0.001	13.9 %	34 (5.2 %)	29 (4.4 %)	3.6 %
Chronic kidney disease	62 (3.4 %)	57 (6.3 %)	<0.001	13.5 %	36 (5.5 %)	32 (4.9 %)	2.8 %
Dialysis	40 (2.2 %)	39 (4.3)	0.01	12.0 %	24 (3.7 %)	25 (3.8 %)	0.8 %
Chronic lung disease	34 (1.9 %)	30 (3.3 %)	0.02	9.2 %	15 (2.3 %)	15 (2.3 %)	0.0 %
Previous malignancy	61 (3.3 %)	146 (16.1 %)	<0.001	44.1 %	52 (8.0 %)	72 (11.0 %)	10.5 %
Atrial fibrillation	48 (2.6 %)	39 (4.3 %)	0.02	9.2 %	24 (3.7 %)	25 (3.8 %)	0.8 %
Estimated GFR (mL/min)	82.8 ± 20.4	74.4 ± 34.3	<0.001	36.2 %	77.4 ± 22.2	77.4 ± 23.5	0.1 %
Mean ejection fraction (%)	58.0 ± 8.6	54.4 ± 11.6	<0.001	36.2 %	55.9 ± 9.9	56.0 ± 10.8	1.0 %
Severe LV dysfunction^a^	42 (2.3 %)	78 (8.6 %)	<0.001	19.8 %	29 (4.4 %)	45 (6.9 %)	8.8 %
**Clinical presentation**							
Chronic coronary syndrome	1372 (74.7 %)	73.4 (80.7 %)	<0.001	14.6 %	509 (77.9 %)	512 (78.4 %)	1.1 %
Acute coronary syndrome	465 (25.3 %)	175 (19.3 %)			144 (22.1 %)	141 (21.6 %)	
**Extent of the diseased vessel**							
1VD	641 (34.9 %)	205 (22.6 %)	<0.001	36.6 %	168 (25.7 %)	172 (26.3 %)	1.4 %
2VD	708 (38.5 %)	318 (35.0 %)			243 (37.2 %)	242 (37.1 %)	
3VD	488 (26.6 %)	386 (42.5 %)			242 (37.1 %)	239 (36.6 %)	
**CTO vessel**							
LAD	810 (44.1 %)	194 (21.3 %)	<0.001	53.8 %	168 (25.7 %)	180 (27.6 %)	4.5 %
LCx	268 (14.6 %)	265 (29.2 %)			146 (22.4 %)	147 (22.5 %)	
RCA	759 (44.1 %)	450 (49.5 %)			339 (51.9 %)	326 (49.9 %)	
**Proximal CTO location**	1528 (83.2 %)	606 (66.7 %)	<0.001	18.5 %	517 (79.2 %)	451 (69.1 %)	10.4 %
**Collateral flow grade (%)**^b^							
0	33 (1.8 %)	51 (5.6 %)	<0.001	37.0 %	15 (2.3 %)	41 (6.3 %)	16.5 %
1	421 (22.9 %)	162 (17.8 %)			119 (18.2 %)	113 (17.3 %)	
2	654 (35.6 %)	356 (39.2 %)			221 (33.8 %)	252 (38.6 %)	
3	729 (39.7 %)	340 (37.4 %)			298 (45.6 %)	247 (37.8 %)	

Values are a mean ± standard deviation or number (%) unless indicated otherwise. Percentages may not total 100 % because of rounding.

The glomerular filtration rate was calculated using CKD-EPI equations.

CTO, chronic total occlusion; GFR, glomerular filtration rate; IQR, interquartile range; LDL, low-density lipoprotein; LV, left ventricle; MI, myocardial infarction; PCI, percutaneous coronary intervention; VD, vessel disease.

a Severe LV dysfunction is defined as a condition where the ejection fraction is less than 30 %.

b Collateral flow grade was not used as a component of propensity score analyses due to difficulty in matching according to the four hierarchical classifications.

All tests were two-sided, and significance was set at P < 0.05. No adjustments were made for multiple testing. Therefore, given the potential for type I errors resulting from multiple comparisons, the observed results should be interpreted as exploratory. All statistical analyses were performed using SAS version 9.4 (SAS Institute, Cary, NC), and intuitive graphs were drawn using R version 3.6.1 (http://www.r-project.org).

## 3 Results

### 3.1 Study population and baseline characteristics

**Fig. 1:**
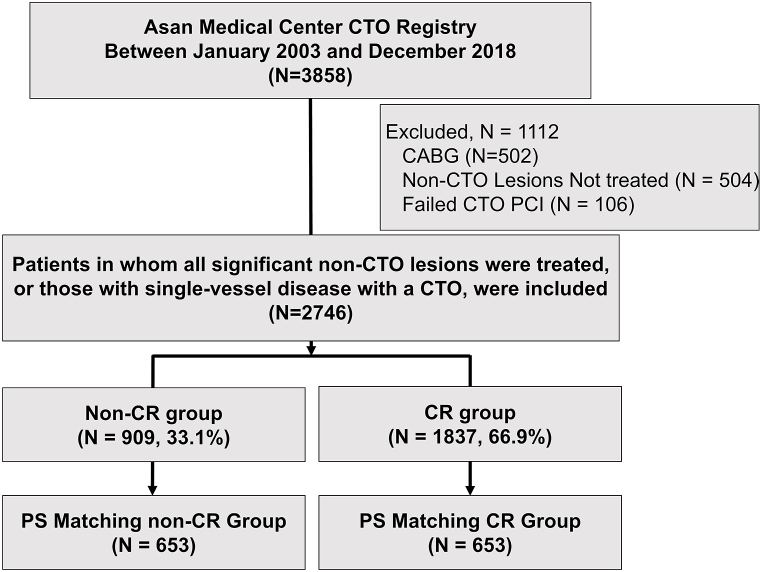
Study flowchart. Patient selection and grouping. CABG, coronary artery bypass graft; CR, complete revascularization; CTO, chronic total occlusion; PCI, percutaneous coronary intervention; PS, propensity score.

A flow diagram of the study cohort is shown in Fig. 1. Of the 3858 patients enrolled in the Asan Medical Center CTO Registry between January 2003 and December 2018, 1112 were excluded from analysis in this study because they underwent CABG, did not undergo revascularization for significant non-CTO lesions, or had failed PCI for CTO lesions. The final cohort consisted of 2746 patients, including 846 with single-vessel CTO and 1900 with multivessel CAD with CTO who also underwent treatment for all significant non-CTO lesions. These patients were stratified into two groups, those who did not achieve CR due to deferred CTO-PCI (n = 909; 33.1 %) and those who achieved CR with CTO-PCI (n = 1837; 66.9 %).

The baseline clinical and anatomic characteristics of the included patients are summarized in Table 1, stratified by the achievement of CR. Prior to propensity score adjustment, significant differences were observed in several baseline characteristics. In general, patients in the non-CR group were older, and had a higher prevalence of comorbidities, a more extensive CAD, decreased renal function, and a lower left ventricular ejection fraction. By contrast, the percentages of patients with left anterior descending artery involvement and proximal CTO lesions were higher in the CR group. The percentages of patients with poor collateral flow were higher in the non-CR group, although the proportions with second- and third-class collateral flow were similar in the two groups.

The distribution of propensity scores in the CR and non-CR groups is indicated in Supplementary Fig. 1. The probability of deferring CTO-PCI was significantly associated with a history of cancer, CTO in the left circumflex artery or right coronary artery, extensive CAD, a history of MI, and an older age (Supplementary Table 1).

Following propensity score adjustment, 653 pairs of patients were successfully matched, resulting in well-balanced baseline covariates in the two groups (Table 1).

### 3.2 Procedural outcomes

The procedural details for both groups are summarized in Supplementary Table 2. Most patients in the CR group underwent revascularization of the CTO lesion via stenting, with 65.9 % of these patients treated with second-generation stents. The target CTO lesion was typically treated with two stents (interquartile range [IQR]: 1.0–2.0), with a median total length of 51 mm (IQR: 33.0–66.0 mm) and a median diameter of 3.1 mm (IQR: 3.0–3.5 mm).

Revascularization of the non-CTO lesions in both groups consisted predominantly of stenting, although there were notable between-group differences in the type, number, diameter, and total length of the DES stents. Even after propensity score matching, procedural differences were found to be attenuated for non-CTO lesions.

### 3.3 Cardiac medication at discharge

Cardiac medications at discharge differed between the CR and non-CR groups. The secondary prevention components of OMT (i.e., beta-blockers, ACE inhibitors/ARBs, statins) were prescribed more frequently in the non-CR group. By contrast, antianginal medications (i.e., calcium channel blockers, nitrates) were prescribed more frequently in the CR group (Supplementary Table 3). The proportions of patients treated with antiplatelet agents were similar in the two groups, although anticoagulation therapy (i.e., NOACs, warfarin) was more frequently prescribed to the non-CR group cases. Following propensity score matching, no significant between-group differences were observed in relation to medications other than calcium channel blockers.

### 3.4 Clinical outcomes in the unmatched population

In the overall population, the median clinical follow-up duration was 5.3 years (interquartile range [IQR]: 3.1–9.6 years): 6.3 years (IQR: 3.4–10.8 years) for the CR group and 4.9 years (IQR: 2.9–5.8 years) for the non-CR group. Due to differences in these follow-up periods, analyses were segmented based on a 5-year follow-up. Throughout the entire follow-up period, 564 primary composite events were recorded (death, n = 405; spontaneous MI, n = 52; stroke, n = 58; any repeat revascularization, n = 284). In addition, there were 297 cardiac deaths and 115 CTO-related repeat revascularizations. The unadjusted event rates of the primary and secondary outcomes, segmented by 5-year and 10-year intervals based on the achievement of CR, are detailed in Supplementary Table 4.

**Fig. 2:**
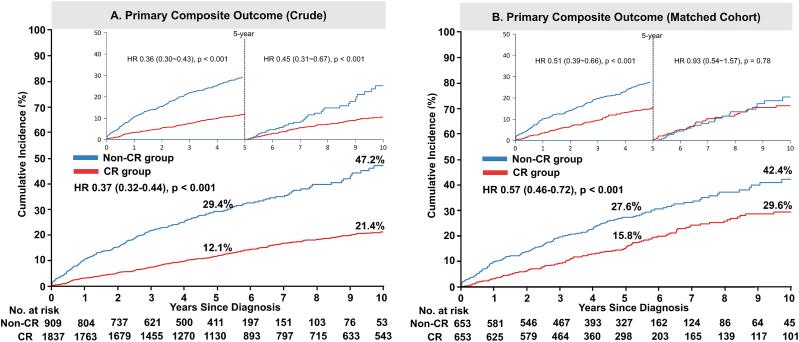
Risks of the primary composite outcome in patients who did and did not achieve complete revascularization. Crude (A) and adjusted (B) event curves for the primary composite outcome, defined as the composite of death from any cause, spontaneous myocardial infarction, stroke, or any repeat revascularization, in the non-CR and CR groups. HRs and 95 % CIs are shown for the CR group versus the non-CR group. CIs, confidence intervals; CR, complete revascularization; HRs, hazard ratios.

Throughout the follow-up duration, the cumulative incidence of the primary composite of death, spontaneous MI, stroke, or any repeat revascularization was significantly lower in the CR group (Table 2 and Fig. 2A). Similar patterns were observed for the individual components of death (i.e., all-cause mortality and cardiac death) (Supplementary Figs. 2A and 2B) or repeat revascularization (i.e., any repeat revascularization and CTO-related repeat revascularization) (Supplementary Figs. 3A and 3B).

**Table 2:** HRs for clinical outcomes during follow-up.

Outcomes	Unadjusted		Propensity score-matched		Propensity score-matched + medication adjusted^b^	
	HR^a^ (95 % CI)	P-Value	HR^a^ (95 % CI)	P-Value	HR^a^ (95 % CI)	P-Value
**Primary composite outcome of death, spontaneous MI, stroke, or any repeat revascularization**^c^						
At 5 years	0.36 (0.30–0.43)	<0.001	0.51 (0.39–0.66)	<0.001	0.52 (0.39–0.70)	<0.001
5–10 years	0.45 (0.31–0.67)	<0.001	0.93 (0.54–1.57)	0.78	NA^b^	NA^b^
At 10 years	0.37 (0.32–0.44)	<0.001	0.57 (0.46–0.72)	<0.001	NA^b^	NA^b^
**All-cause mortality**						
At 5 years	0.33 (0.26–0.41)	<0.001	0.60 (0.44–0.82)	0.01	0.65 (0.45–0.93)	0.02
5–10 years	0.41 (0.26–0.62)	<0.001	0.93 (0.52–1.67)	0.81	NA^b^	NA^b^
At 10 years	0.34 (0.28–0.42)	<0.001	0.66 (0.51–0.87)	0.01	NA^b^	NA^b^
**Any repeat revascularization**						
At 5 years	0.75 (0.57–0.99)	0.04	0.77 (0.53–1.12)	0.17	0.68 (0.45–1.03)	0.07
5–10 years	0.43 (0.26–0.74)	0.01	0.40 (0.18–0.86)	0.02	NA^b^	NA^b^
At 10 years	0.67 (0.53–0.86)	0.01	0.67 (0.48–0.95)	0.02	NA^b^	NA^b^
**Cardiac death**						
At 5 years	0.31 (0.23–0.40)	<0.001	0.56 (0.38–0.82)	0.01	0.61 (0.39–0.94)	0.02
5–10 years	0.37 (0.23–0.59)	<0.001	0.87 (0.46–1.64)	0.67	NA^b^	NA^b^
At 10 years	0.32 (0.26–0.41)	<0.001	0.63 (0.46–0.88)	0.01	NA^b^	NA^b^
**CTO-related repeat revascularization**						
At 5 years	0.45 (0.30–0.68)	<0.001	0.43 (0.24–0.78)	0.01	0.42 (0.22–0.76)	0.01
5–10 years	0.35 (0.15–0.81)	0.01	0.50 (0.16–1.56)	0.23	NA^b^	NA^b^
At 10 years	0.43 (0.30–0.62)	<0.001	0.44 (0.27–0.74)	0.01	NA^b^	NA^b^

CI, confidence interval; MI, myocardial infarction; NA, not available.

a HRs are for the PCI group compared with the OMT group.

b HR was calculated up to 5 years under the assumption that discharge medication could have an effect for this period; values after 5 years were not estimated.

c HR for spontaneous MI and stroke could not be calculated because of the small number of events.

### 3.5 Clinical outcomes in the matched population

**Fig. 3:**
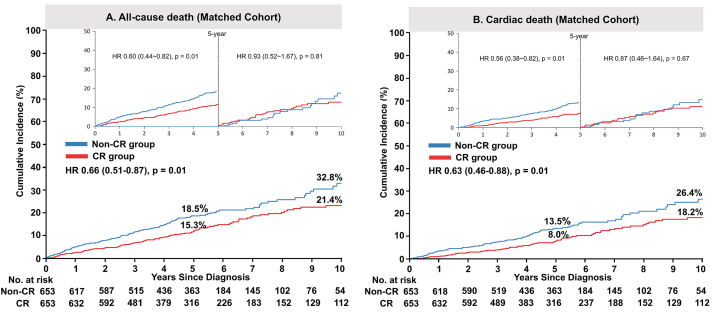
Risks of all-cause or cardiac death in patients who did and did not achieve complete revascularization. Adjusted event curves for all-cause mortality (A) and cardiac death (B) in the non-CR and CR groups. HRs and 95 % CIs are shown for the CR group versus the non-CR group. CIs, confidence intervals; CR, complete revascularization; HRs, hazard ratios.

**Fig. 4:**
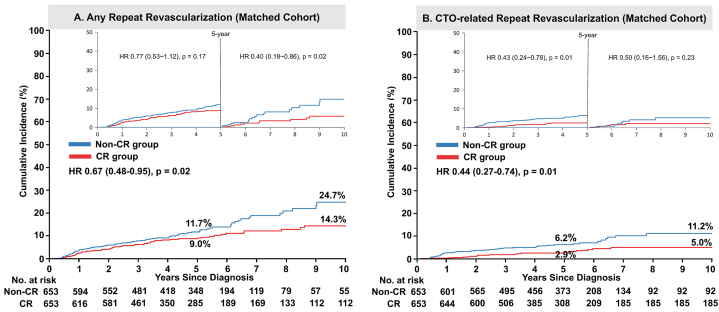
Risks of repeat revascularization in patients who did and did not achieve complete revascularization. Adjusted event curves for any repeat revascularization (A) and CTO-related repeat revascularization (B) in the non-CR and CR groups. HRs and 95 % CIs are shown for the CR group versus the non-CR group. CIs, confidence intervals; CR, complete revascularization; CTO, chronic total occlusion; HRs, hazard ratios.

In the propensity-matched cohort, differences in the primary outcome between the two groups decreased but the adjusted risk was significantly lower in the CR group (hazard ratio [HR]: 0.57; 95 % confidence interval [CI]: 0.46–0.72; P < 0.001) (Table 2 and Fig. 2B). The adjusted risks for all-cause mortality (HR: 0.66; 95 % CI: 0.51–0.87; P = 0.003) and cardiac death (HR: 0.63; 95 % CI: 0.46–0.88; P = 0.007) were also significantly lower in the CR group (Table 2 and Fig. 3A and B). Similarly, the risk of repeat revascularization was lower in the CR group, both for any repeat revascularization (HR: 0.67; 95 % CI: 0.48–0.95; P = 0.023) and specifically for CTO-related repeat revascularization (HR: 0.44; 95 % CI: 0.27–0.74; P = 0.002) (Table 2 and Fig. 4A and B).

Notably, the HR for repeat revascularization varied over time. The between-group difference in the rate of any repeat revascularization was more pronounced during the last 5 years, whereas the difference in the rate of CTO-related repeat revascularization was more pronounced during the first 5 years.

### 4.6 Subgroup analyses in the matched population

**Fig. 5:**
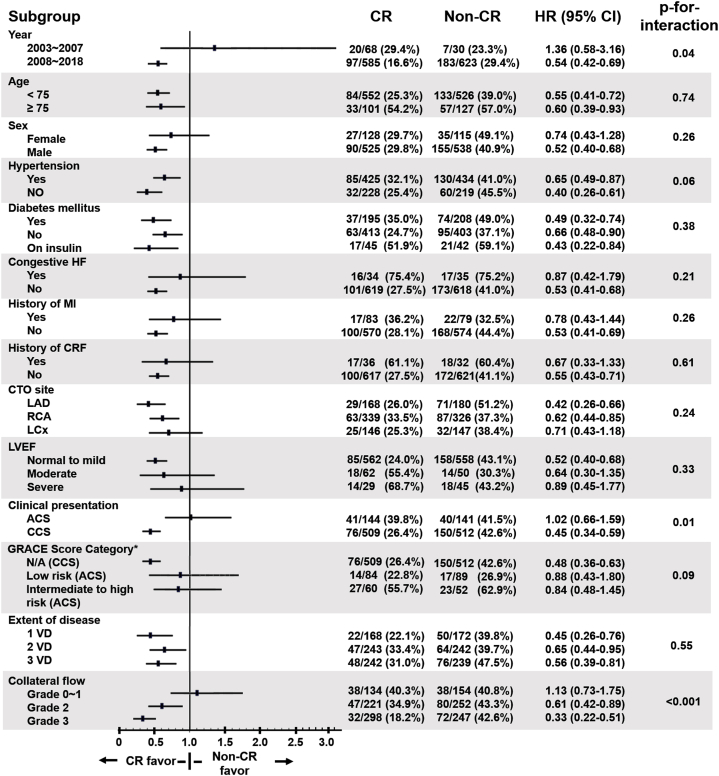
Ten-year rates of the primary composite outcome in patient subgroups. HRs for the 10-year rate of the primary composite outcome, defined as the composite of death from any cause, spontaneous myocardial infarction, stroke, or any repeat revascularization, as determined by Cox regression analysis in subgroups of patients with CTO in the non-CR and CR groups. HRs and 95 % CIs are shown for the CR group versus the non-CR group. ACS, acute coronary syndrome; CCS, chronic coronary syndrome; CIs, confidence intervals; CRF, chronic renal failure; GRACE, Global Registry of Acute Coronary Events; HF, heart failure; HRs, hazard ratios; LAD, left anterior descending artery; LCx, left circumflex coronary artery; LVEF, left ventricular ejection fraction; MI, myocardial infarct; N/A, not applicable; RCA, right coronary artery; VD, vessel disease. ∗ Patients were categorized as: N/A (CCS), low risk (ACS), and intermediate to high risk (ACS), based on GRACE scores, to analyze outcomes in both ACS and CCS patients (GRACE score not applicable to CCS).

Cox regression analysis was performed to assess the consistency of CR benefit across subgroups, comparing the incidence of primary composite outcomes between CR and non-CR groups. The beneficial effect of CR remained consistent across various cardiovascular risk factors, including age, hypertension, diabetes, and the extent of CAD (from single to triple-vessel disease). A significant interaction was observed with clinical presentation, showing greater benefit in chronic coronary syndrome (CCS) compared to acute coronary syndrome (ACS). This beneficial impact persisted throughout the study period from 2003 and was more pronounced in patients with well-developed collaterals (Fig. 5).

### 4.7 Independent predictors of clinical outcomes

The independent predictors of the primary composite outcome, its individual components, and repeat revascularization are summarized in Table 3. In the overall population, CTO-PCI was independently associated with the primary composite outcome (HR: 0.56; 95 % CI: 0.47–0.68; P < 0.001), all-cause mortality (HR: 0.54; 95 % CI: 0.43–0.67; P < 0.001) and repeat revascularization (HR: 0.72; 95 % CI: 0.56–0.93; P = 0.012). The primary composite outcome was also significantly associated with age, diabetes, previous PCI, previous stroke, chronic renal failure, malignancy, lower ejection fraction, and more extensive CAD. Despite some differences in the magnitude of the HRs and their corresponding P-values, most of the major correlates of the 10-year clinical outcomes in the overall population remained significant correlates in each group (Supplementary Table 5).

**Table 3:** Independent predictors of clinical outcomes in the overall population.

	Overall	
	HR^a^ (95 % CI)	P-Value
Primary composite outcome		
CTO-PCI	0.56 (0.47–0.68)	<0.001
Age >75 years	1.94 (1.59–2.36)	<0.001
Diabetes mellitus	1.34 (1.11–1.60)	0.01
Diabetes mellitus requiring insulin	1.53 (1.14–2.06)	0.01
Previous PCI	1.23 (1.03–1.48)	0.02
Previous stroke	1.48 (1.16–1.89)	0.01
Chronic renal failure	2.28 (1.69–3.06)	<0.001
History of cancer	2.12 (1.66–2.71)	<0.001
Severe LV dysfunction^b^	2.53 (1.89–3.40)	<0.001
Disease extent (3 vessel disease)^c^	1.29 (1.04–1.59)	0.02
**All-cause mortality**		
CTO-PCI	0.54 (0.43–0.67)	<0.001
Age >75 years	2.48 (1.99–3.09)	<0.001
Diabetes mellitus	1.60 (1.31–1.95)	<0.001
Previous stroke	1.62 (1.23–2.14)	0.01
Chronic renal failure	2.94 (2.15–4.02)	<0.001
History of cancer	2.38 (1.82–3.13)	<0.001
Severe LV dysfunction^b^	3.33 (2.40–4.61)	<0.001
**Any repeat revascularization**		
CTO-PCI	0.72 (0.56–0.93)	0.01
Previous PCI	1.58 (1.23–2.02)	<0.001
Disease extent (3 vessel disease)^c^	2.23 (1.63–3.05)	<0.001
CTO site (LCx)^d^	0.65 (0.45–0.94)	0.02
**Cardiac death**		
CTO-PCI	0.49 (0.38–0.63)	<0.001
Age >75	3.16 (2.46–4.05)	<0.001
Diabetes mellitus	1.88 (1.49–2.37)	<0.001
Congestive heart failure	1.71 (1.14–2.56)	0.01
Previous stroke	1.90 (1.40–2.58)	<0.001
Chronic renal failure	2.98 (2.10–4.23)	<0.001
Severe LV dysfunction^b^	3.07 (2.08–4.55)	<0.001
Disease extent (3 vessel disease)^c^	1.44 (1.07–1.94)	0.02
**CTO lesion-related revascularization**		
CTO-PCI	0.37 (0.25–0.54)	<0.001
CTO site (LCx)^d^	0.42 (0.24–0.75)	0.01

CI, confidence interval; CTO, chronic total occlusion; LAD, left anterior descending; LCx, left circumflex coronary artery; LV, left ventricle; MI, myocardial infarction; NA, not available; PCI, percutaneous coronary intervention.

a HRs are for the PCI group compared with the OMT group.

b Risk of severe LV dysfunction relative to normal LV function.

c Risk of 3-vessel disease relative to 1-vessel disease.

d Risk of CTO site (LCx) relative to CTO site (LAD).

## 4 Discussion

We have conducted a comprehensive analysis of a large, real-world cohort of patients with coronary CTO. After excluding 1112 of 3858 CTO patients (CABG, unsuccessful CTO-PCI, or untreated significant multivessel disease), we compared the baseline characteristics and long-term outcomes between patients with and without CR achieved through CTO-PCI. The results of these analyses indicated that the clinical and angiographic characteristics differed significantly between the CR and non-CR groups, with the rates of cardiovascular risk factors being higher in the non-CR group. Following propensity score matching of patients in the CR and non-CR groups, the CR group showed significantly lower rates of major adverse cardiovascular events, especially rates of death and repeat revascularization. Moreover, CR showed significant benefits in all patient subgroup analyses based on major cardiovascular disease risk factors.

Successful recanalization of a CTO is thought to result in an improvement in regional wall motion, particularly in the hibernating myocardium [14]. This may enhance not only patient quality of life and angina relief, but may reduce the risk of major adverse cardiovascular events and improve survival. Although RCTs and meta-analyses have indicated that CTO-PCI does not significantly improve survival compared with OMT [15], those prior studies had limitations, including small sample sizes, short study durations of <5 years, and a focus on low-risk patients. In addition, the high crossover rate from CTO-OMT to CTO-PCI and complications from failed CTO-PCI may have contributed to CTO-OMT appearing more beneficial than expected. To overcome these limitation issues, the present study evaluated the long-term outcomes of CTO-PCI in a real-world patient population. More precisely, the effect of CR with CTO-PCI was assessed by focusing on patients with single-vessel CTO or who underwent revascularization for all significant non-CTO lesions, while excluding those with unsuccessful PCI for CTO lesions.

Patients with a deferred CTO-PCI who did not achieve CR had higher rates of cardiovascular comorbidities and more complex anatomy than patients who achieved CR. Unadjusted analyses showed that cardiovascular risk was higher in patients in the non-CR group. Even after rigorous control for baseline characteristics and confounders using propensity score matching, the rates of major adverse cardiovascular events and mortality were significantly higher in the non-CR cases. The present study findings are that the benefits of CR with CTO-PCI consisted primarily of lower mortality or cardiac mortality rates, suggesting that the relief of myocardial ischemia achieved by PCI enhances the long-term prognosis. The benefit of CR showed differential effects based on clinical presentation, with a significant effect in chronic coronary syndrome (CCS) but not in acute coronary syndrome (ACS). This disparity might be explained by ACS presentations being driven by factors beyond the CTO itself, such as plaque instability in non-CTO lesions or inflammatory burden, which could attenuate the benefit of CTO revascularization. Furthermore, even when stratified by GRACE score in ACS patients, CR achieved through CTO-PCI after treating the culprit lesion did not significantly impact clinical outcomes. Nevertheless, these benefits of CR were consistently observed across different subgroups, irrespective of the presence of major cardiovascular risk factors such as older age, hypertension, diabetes, and advanced CAD. Importantly, patients achieving CR through CTO-PCI after 2008 showed significantly better clinical outcomes along with advances in DES technology and widespread use of potent antiplatelet and statin therapy, suggesting the enhanced value of CR in the contemporary era of advanced CTO techniques. The favorable outcomes may be particularly attributed to the restoration of antegrade flow in viable but ischemic myocardium in patients with well-developed collaterals, suggesting the importance of collateral-dependent myocardial viability in identifying optimal candidates for revascularization [16]. In our study, we also observed that higher collateral flow grades were associated with better clinical outcomes, further supporting the significance of collateral circulation in the prognosis of CTO patients. Achievement of CR with CTO-PCI thus appears to be an important prognostic determinant in itself, suggesting the need to reevaluate the role of PCI for CTO.

Patients with coronary CTOs have a high atherosclerotic burden [17]. CTO lesions can be considered surrogate markers of advanced cardiovascular disease rather than just local coronary lesions. Unlike previous studies that highlighted the limited role of PCI [18], we observed that achieving CR with CTO-PCI was associated with improvements in prognosis, including better survival rates and a reduction in repeat revascularization rates compared with the non-CR group. While crude analysis demonstrated sustained benefits of CR, propensity score matching revealed that mortality benefit was limited to the first 5 years, reflecting the progressive nature of atherosclerosis. Interestingly, repeat revascularization showed a different pattern, with rates diverging after 5 years. This temporal difference reflects the distinct nature of hard clinical outcomes versus symptom-driven reinterventions in CTO patients. The differences in medical treatment between groups may have influenced these long-term patterns. The CR group received more anti-anginal medications for symptom control, while the non-CR group used more secondary prevention medications. This differential approach to medical therapy could explain both the convergence of hard outcomes in later years and the reduced need for angiographic evaluations in the CR group.

The present study had several limitations of note. First, as a nonrandomized observational study, it was susceptible to potential selection and ascertainment biases. Despite rigorous adjustment for baseline clinical risk factors and non-CTO lesion characteristics, unmeasured confounders, such as patient frailty or a more detailed assessment of atherosclerotic burden, may have influenced our results. Second, the difference in follow-up time between the CR and non-CR groups may have led to an overestimation in the comparison of outcomes over the last 5 years. Third, we did not systematically collect comprehensive data on long-term medication use and adherence to guideline-directed medical management after initial treatment, which may have varied significantly over time. Fourth, there was no independent angiographic core laboratory assessment, with procedural success being determined by operator evaluation of TIMI 3 flow and residual stenosis <30 %, without systematic assessment of side branch preservation. Finally, the study patients were not quantitatively stratified by the extent to which collateral flow relieved ischemic burden in the CTO territory. Because the relief of ischemic burden after treatment is not uniform across patients, further large-scale studies are needed to accurately determine the prognostic value of recanalization in CTO, particularly in relation to the specific ischemic burden within the CTO territory.

## 5 Conclusions

In conclusion, evaluations of a real-world registry of patients with CTO has revealed that achieving CR with CTO-PCI is significantly associated with clinical benefits, including the primary composite outcome of death, spontaneous MI, stroke, or repeat revascularization. This association persists even after adjustment for clinical covariates. The clinical benefits of CR with CTO-PCI includes both improved survival and a reduction in CTO-related repeat revascularization for symptom management. Furthermore, the benefits of revascularization remain consistent across patient subgroups, independent of major cardiovascular risk factors. Additional studies are required to reevaluate the role of PCI in the management of patients with CTO, especially long-term clinical outcomes.

## CRediT authorship contribution statement

**Tae Oh Kim:** Writing – original draft, Visualization, Investigation. **SeHee Kim:** Formal analysis. **Min-Ju Kim:** Formal analysis. **Do-Yoon Kang:** Writing – review & editing, Methodology. **Pil Hyung Lee:** Writing – review & editing, Resources, Project administration, Conceptualization. **Soo-Jin Kang:** Writing – review & editing, Methodology. **Cheol Whan Lee:** Writing – review & editing, Methodology. **Young-Hak Kim:** Writing – review & editing, Methodology. **Jong-Young Lee:** Writing – review & editing, Methodology. **Seung-Whan Lee:** Writing – review & editing, Writing – original draft, Visualization, Supervision, Data curation, Conceptualization.

## Declaration of competing interest

This study was supported by a grant (2024IE0006) from the 10.13039/501100005006Asan Institute for Life Sciences, Asan Medical Center. The funding source had no involvement in the design of the study, the collection, analysis, and interpretation of data, the writing of the manuscript, or the decision to submit the paper for publication. The authors have no conflicts of interest to declare.
