# Supplementary material for: Long-term impacts of complete revascularization on clinical outcomes in patients with coronary chronic total occlusion
Source: Heliyon. 2024 Nov 12;10(23):e40326. doi: 10.1016/j.heliyon.2024.e40326 (PMC11625259; doi:10.1016/j.heliyon.2024.e40326)
Supplement: Multimedia component 1 [file mmc1.docx]

**Supplementary Appendix**

This appendix provides data that is supplemental to that in TO Kim et al. “Long-term Impacts of Complete Revascularization on Clinical Outcomes in Patients with Coronary Chronic Total Occlusion”

**Supplementary Table 1.** Clinical Variables that Led to a CTO-PCI Deferral Among the Patients

**Supplementary Table 2.** Procedural Characteristics in the Patients with CTO Who Did or Did Not Achieve Complete Revascularization

**Supplementary Table 3.** Cardiac-Related Medications at Discharge in the Patients with CTO Who Did and Did Not Achieve Complete Revascularization

**Supplementary Table 4.** Unadjusted Event Rates of the Primary and Secondary Outcomes in the Patients with CTO Who Did and Did Not Achieve Complete Revascularization

**Supplementary Table 5.** Independent Predictors of Clinical Outcomes in Each Treatment Group

**Supplementary Figure 1.** Propensity Scores for OMT

**Supplementary Figure 2.** Kaplan–Meier Analyses of All-Cause Death and Cardiac Death in the Unadjusted Patient Population

**Supplementary Figure 3.** Kaplan–Meier Analyses of the Individual Components of Repeat Revascularization in the Unadjusted Patient Population

**Supplementary Table 1.** Clinical Variables that Led to a CTO-PCI Deferral Among the Patients

| **Variable** | **Estimate** | **Standard Error** | **p value** |
| --- | --- | --- | --- |
| History of cancer | 1.5263 | 0.1824 | < 0.001 |
| CTO site (LCx) | 1.4958 | 0.1401 | < 0.001 |
| CTO site (RCA) | 1.0063 | 0.1166 | < 0.001 |
| Disease extent (3 vessel disease) | 0.8869 | 0.1323 | < 0.001 |
| Previous MI | 0.5703 | 0.1618 | < 0.001 |
| Hyperlipidemia | 0.4384 | 0.1318 | < 0.001 |
| Year of procedure (late year) | 0.1235 | 0.013 | < 0.001 |
| Age (> 75 years) | 0.0341 | 0.00609 | < 0.001 |
| Diabetes mellitus | 0.2696 | 0.1081 | 0.0126 |
| Chronic coronary syndrome | 0.2517 | 0.1204 | 0.0367 |
| Hypertension | 0.2002 | 0.108 | 0.0638 |
| Previous stroke | 0.2884 | 0.1696 | 0.0891 |
| Peripheral artery disease | 0.262 | 0.2428 | 0.2807 |
| Dialysis | 0.2362 | 0.4607 | 0.6082 |
| Disease extent (2 vessel disease) | 0.1914 | 0.1231 | 0.12 |
| Current smoker | 0.1519 | 0.1166 | 0.1926 |
| Chronic lung disease | 0.1177 | 0.3092 | 0.7035 |
| Previous PCI | 0.1068 | 0.1184 | 0.3674 |
| Atrial fibrillation | 0.0145 | 0.2659 | 0.9566 |
| Sex, male | 0.00905 | 0.1331 | 0.9458 |
| Estimated GFR | -0.00417 | 0.00337 | 0.2157 |
| Diabetes mellitus requiring insulin | -0.00745 | 0.2182 | 0.9728 |
| BMI | -0.0284 | 0.0157 | 0.071 |
| Severe LV dysfunction | -0.0342 | 0.00549 | < 0.001 |
| Congestive heart failure | -0.2928 | 0.2641 | 0.2676 |
| Chronic renal failure | -0.3243 | 0.3904 | 0.4061 |

**Supplementary Table 2.** Procedural Characteristics in the Patients with CTO Who Did and Did Not Achieve Complete Revascularization*.

| **Variable** | **Unadjusted** | | | **Propensity Score-Matched** | | |
| --- | --- | --- | --- | --- | --- | --- |
|  | **CR (N=1837)** | **Non-CR (N=909)** | **p Value** | **CR (N=653)** | **Non-CR (N=653)** | **p Value** |
| **CTO lesion treatment**^†^ | 1837 (100%) |  |  | 653 (100%) |  |  |
| Ballooning | 91 (5.0%) |  |  | 47 (7.2%) |  |  |
| Stenting | 1746 (95.0%) |  |  | 606 (92.8%) |  |  |
| **Stent generation for CTO**^‡^ |  |  |  |  |  |  |
| BMS | 31 (1.7%) |  |  | 11 (1.7%) |  |  |
| 1^st^ DES | 505 (27.5%) |  |  | 71 (10.9%) |  |  |
| 2^nd^ DES | 1210 (65.9%) |  |  | 524 (80.2%) |  |  |
| **No. of stents for CTO** | 2.0 (1.0–2.0) |  |  | 2.0 (1.0–2.0) |  |  |
| **Stent diameter for CTO, mm** | 3.1 (3.0–3.5) |  |  | 3.1 (3.0–3.5) |  |  |
| **Total stent length for CTO, mm** | 51.0 (33.0–66.0) |  |  | 53.0 (33.0–74.0) |  |  |
| **IVUS guided PCI** | 1745 (94.9%) |  |  | 614 (94.1%) |  |  |
| **Non-CTO lesion treatment** | 1,196 (65.1%) | 704 (77.4%) |  | 485 (74.3%) | 481 (73.7%) |  |
| Ballooning | 1 (0.1%)* | 13 (1.4%)* | <0.001 | 0 | 10 (1.5%) | 0.004 |
| Stenting | 1,195 (65.1%)* | 691 (76.1%)* |  | 485 (74.3%) | 471 (72.1%) |  |
| **Stent generation for Non-CTO** |  |  |  |  |  |  |
| BMS | 21 (1.1%)* | 6 (0.7%)* | <0.001 | 12 (1.8%) | 6 (0.9%) | 0.368 |
| 1^st^ DES | 302 (16.4%)* | 72 (7.9%) |  | 67 (10.3%) | 62 (9.5%) |  |
| 2^nd^ DES | 872 (47.5%)* | 613 (67.4%)* |  | 406 (62.2%) | 413 (63.2%) |  |
| **No. of stents for non-CTO** | 1.0 (1.0–2.0) | 1.0 (1.0–2.0) | 0.001 | 1.0 (1.0~2.0) | 1.0 (1.0–2.0) | 0.001 |
| **Stent diameter for non-CTO, mm** | 3.25 (3.0–3.5) | 3.5 (3.0–3.5) | 0.003 | 3.3 (3.0~3.5) | 3.5 (3.0–3.5) | 0.012 |
| **Total stent length for non-CTO, mm** | 32.0 (23.0–40.0) | 38.0 (24.0–52.0) | 0.002 | 33.0 (23.0–45.0) | 36 (23.3–52.0) | 0.03 |

Values are numbers (%) or a median (interquartile range).

*Percentage of total patients in each group.

BMS, bare-metal stent; CABG, coronary artery bypass grafting; CTO, chronic total occlusion; DES, drug-eluting stent; IVUS, intravascular ultrasound; PCI, percutaneous coronary intervention.

**Supplementary Table 3.** Cardiac-Related Medications at Discharge in the Patients with CTO Who Did and Did Not Achieve Complete Revascularization

|  | **Unadjusted** | | | **Propensity Score-Matched** | | |
| --- | --- | --- | --- | --- | --- | --- |
|  | **CR (N=1837)** | **Non-CR (N=909)** | **p Value** | **CR (N=653)** | **Non-CR (N=653)** | **p Value** |
| Aspirin | 1764 (96.0%) | 863 (94.9%) | 0.224 | 622 (95.3%) | 621 (95.1%) | 1.00 |
| Clopidogrel | 1726 (94.0%) | 856 (94.2%) | 0.893 | 605 (92.6%) | 603 (92.3%) | 0.916 |
| P2Y12 inhibitor | 1782 (97.0%) | 864 (95.0%) | 0.014 | 617 (94.5%) | 614 (94.1%) | 0.812 |
| Dual antiplatelet therapy | 1778 (96.8%) | 863 (94.9%) | 0.023 | 614 (94.0%) | 612 (93.7%) | 0.908 |
| NOAC or warfarin | 48 (2.6%) | 39 (4.3%) | 0.018 | 24 (3.7%) | 25 (3.8%) | 0.8 |
| Beta blocker | 1187 (64.6%) | 674 (74.1%) | <0.001 | 463 (70.9%) | 486 (74.4%) | 0.172 |
| Calcium channel blocker | 1259 (68.5%) | 576 (63.4%) | 0.007 | 470 (72.0%) | 425 (65.1%) | 0.009 |
| ACE inhibitor/ ARB | 677 (36.9%) | 469 (51.6%) | <0.001 | 284 (43.5%) | 325 (49.8%) | 0.027 |
| Nitrate | 664 (36.1%) | 260 (28.6%) | <0.001 | 177 (27.1%) | 182 (27.9%) | 0.804 |
| Statin | 1503 (81.8%) | 826 (90.9%) | <0.001 | 600 (91.9%) | 595 (91.1%) | 0.691 |

Values are numbers (%).

ACE, angiotensin-converting enzyme; ARB, angiotensin II receptor blocker.

**Supplementary Table 4.** Unadjusted Event Rates of the Primary and Secondary Outcomes in the Patients with CTO Who Did and Did Not Achieve Complete Revascularization

|  | **Unadjusted** | | | | **Propensity Score-Matched** | | | |
| --- | --- | --- | --- | --- | --- | --- | --- | --- |
|  | **Overall**  **(n=2746)** | **CR**  **(n=1837)** | **Non-CR (n=909)** | **p Value** | **Overall**  **(n=1306)** | **CR**  **(n=653)** | **Non-CR**  **(n=653)** | **p Value** |
| **Primary composite of all-cause death, spontaneous MI, stroke, or any repeat revascularization** |  |  |  |  |  |  |  |  |
| 5-year | 439 (17.8%) | 197 (12.1%) | 242 (29.4%) | <0.001 | 250 (21.9%) | 85 (15.8%) | 165 (27.6%) | <0.001 |
| 5–10 years | 125 (12.6%) | 89 (10.6%) | 36 (25.2%) | <0.001 | 57 (17.8%) | 32 (16.4%) | 25 (20.5%) | 0.777 |
| 10-year | 564 (28.1%) | 286 (21.4%) | 278 (47.2%) | <0.001 | 307 (35.8%) | 117 (29.6%) | 190 (42.4%) | <0.001 |
| **All-cause death** |  |  |  |  |  |  |  |  |
| 5-year | 307 (12.6%) | 129 (8.0%) | 178 (22.0%) | <0.001 | 170 (15.3%) | 62 (15.3%) | 108 (18.5%) | 0.001 |
| 5–10 years | 98 (9.5%) | 67 (7.7%) | 31 (21.2%) | <0.001 | 47 (14.3%) | 26 (12.8%) | 21 (17.5%) | 0.811 |
| 10-year | 405 (20.9%) | 196 (15.0%) | 209 (38.5%) | <0.001 | 217 (27.4%) | 88 (23.0%) | 129 (32.8%) | 0.003 |
| **Spontaneous MI** |  |  |  |  |  |  |  |  |
| 5-year | 42 (1.8%) | 21 (1.3%) | 21 (2.7%) | 0.009 | 28 (2.5%) | 9 (1.7%) | 19 (3.3%) | 0.075 |
| 5–10 years | 10 (1.0%) | 10 (1.2%) | 0 (0.0%) | 0.162 | 2 (0.8%) | 2 (1.3%) | 0 (0.0%) | NA^*^ |
| 10-year | 52 (2.8%) | 31 (2.5%) | 21 (2.7%) | 0.052 | 30 (3.3%) | 11 (3.0%) | 19 (3.3%) | 0.143 |
| **Stroke** |  |  |  |  |  |  |  |  |
| 5-year | 49 (2.0%) | 22 (1.4%) | 27 (3.4%) | <0.001 | 33 (2.9%) | 8 (1.4%) | 25 (4.3%) | 0.006 |
| 5–10 years | 9 (1.0%) | 7 (0.8%) | 2 (2.3%) | 0.579 | 1 (0.5%) | 0 (0.0%) | 1 (1.4%) | NA^*^ |
| 10-year | 58 (3.0%) | 29 (2.2%) | 29 (5.6%) | <0.001 | 34 (3.4%) | 8 (1.4%) | 26 (5.6%) | 0.004 |
| **Any repeat revascularization** |  |  |  |  |  |  |  |  |
| 5-year | 217 (9.2%) | 135 (8.2%) | 82 (11.2%) | 0.039 | 112 (10.4%) | 48 (9.0%) | 64 (11.7%) | 0.172 |
| 5–10 years | 67 (7.4%) | 47 (6.3%) | 20 (12.9%) | 0.001 | 29 (9.5%) | 10 (5.8%) | 19 (14.8%) | 0.019 |
| 10-year | 284 (15.8%) | 182 (14.0%) | 102 (22.7%) | 0.002 | 141 (18.9%) | 58 (14.3%) | 83 (24.7%) | 0.023 |
| **Cardiac death** |  |  |  |  |  |  |  |  |
| 5-year | 214 (9.1%) | 87 (5.5%) | 127 (16.6%) | <0.001 | 115 (10.8%) | 40 (8.0%) | 75 (13.5%) | 0.003 |
| 5–10 years | 83 (8.1%) | 55 (6.4%) | 28 (17.9%) | <0.001 | 41 (12.5%) | 22 (11.2%) | 19 (14.9%) | 0.673 |
| 10-year | 297 (16.4%) | 142 (11.5%) | 155 (31.5%) | <0.001 | 156 (22.0%) | 62 (18.2%) | 94 (26.4%) | 0.007 |
| **CTO-related repeat revascularization** |  |  |  |  |  |  |  |  |
| 5-year | 90 (3.8%) | 45 (2.8%) | 45 (6.0%) | <0.001 | 50 (4.6%) | 15 (2.9%) | 35 (6.2%) | 0.005 |
| 5–10 years | 25 (2.4%) | 16 (1.9%) | 9 (5.1%) | 0.01 | 13 (3.5%) | 5 (2.2%) | 8 (5.3%) | 0.232 |
| 10-year | 115 (6.1%) | 61 (4.6%) | 54 (10.8%) | <0.001 | 63 (7.9%) | 20 (5.0%) | 43 (11.2%) | 0.002 |

Data are numbers (%).

^*^P-values for spontaneous MI and stroke in the two groups after propensity score matching could not be calculated due to low outcome rates.

CI, confidence interval; MI, myocardial infarction; PCI, percutaneous coronary intervention; CABG, coronary artery bypass grafting.

**Supplementary Table 5.** Independent Predictors of Clinical Outcomes in Each Treatment Group

|  | **Non-CR** | | **CR** | | **p-for -interaction** |
| --- | --- | --- | --- | --- | --- |
|  | **HR (95% CI)** | **p Value** | **HR (95% CI)** | **p Value** |  |
| **Primary composite outcome*** |  |  |  |  |  |
| CTO-PCI |  |  |  |  |  |
| Age >75 years | 1.83 (1.42–2.36) | <0.001 | 2.93 (2.20–3.91) | <0.001 | 0.017 |
| Diabetes mellitus | 1.82 (1.44–2.30) | <0.001 | 1.50 (1.18–1.91) | 0.001 | 0.262 |
| Diabetes mellitus requiring insulin | 2.19 (1.51–3.17) | <0.001 | 2.52 (1.72–3.70) | <0.001 | 0.601 |
| Previous PCI | 1.07 (0.84–1.38) | 0.584 | 1.36 (1.06–1.75) | 0.017 | 0.188 |
| Previous stroke | 1.57 (1.13–2.17) | 0.007 | 2.19 (1.54–3.11) | <0.001 | 0.174 |
| Chronic renal failure | 2.45 (1.67–3.57) | <0.001 | 4.43 (2.97–6.59) | <0.001 | 0.034 |
| History of cancer | 2.34 (1.79–3.06) | <0.001 | 1.94 (1.13–3.32) | 0.016 | 0.544 |
| Severe LV dysfunction | 2.06 (1.43–2.96) | <0.001 | 4.59 (2.86–7.37) | <0.001 | 0.045 |
| Disease extent (3 vessel disease) | 1.33 (0.98–1.79) | 0.069 | 1.53 (1.14–2.04) | 0.004 | 0.787 |
| **All-cause death** |  |  |  |  |  |
| CTO-PCI |  |  |  |  |  |
| Age >75 years | 2.28 (1.72–3.02) | <0.001 | 3.90 (2.83–5.38) | <0.001 | 0.014 |
| Diabetes mellitus | 1.70 (1.30–2.23) | <0.001 | 1.89 (1.42–2.51) | <0.001 | 0.605 |
| Previous stroke | 1.84 (1.29–2.62) | 0.001 | 2.35 (1.56–3.55) | <0.001 | 0.377 |
| Chronic renal failure | 3.03 (2.00–4.57) | <0.001 | 5.42 (3.50–8.38) | <0.001 | 0.057 |
| History of cancer | 2.91 (2.17–3.92) | <0.001 | 2.32 (1.26–4.27) | 0.007 | 0.511 |
| Severe LV dysfunction | 2.85 (1.93–4.21) | <0.001 | 5.38 (3.14–9.21) | <0.01 | 0.266 |
| **Spontaneous MI** |  |  |  |  |  |
| NA^*^ |  |  |  |  |  |
| **Stroke** |  |  |  |  |  |
| NA^*^ |  |  |  |  |  |
| **Any repeat revascularization** |  |  |  |  |  |
| CTO-PCI |  |  |  |  |  |
| Previous PCI | 1.42 (0.96–2.12) | 0.082 | 1.48 (1.09–2.03) | 0.013 | 0.876 |
| Disease extent (3 vessel disease) | 1.35 (0.83–2.22) | 0.231 | 2.58 (1.75–3.82) | <0.001 | 0.083 |
| CTO site (LCx) |  |  |  |  |  |
| **Cardiac death** |  |  |  |  |  |
| CTO-PCI |  |  |  |  |  |
| Age >75 years | 2.76 (2.01–3.81) | <0.001 | 4.08 (2.81–5.94) | <0.001 | 0.121 |
| Diabetes mellitus | 2.38 (1.73–3.29) | <0.001 | 1.80 (1.29–2.52) | 0.001 | 0.237 |
| Congestive heart failure | 2.84 (1.75–4.59) | <0.001 | 7.22 (4.40–11.9) | <0.001 | 0.008 |
| Previous stroke | 2.25 (1.53–3.33) | <0.001 | 2.69 (1.69–4.27) | <0.001 | 0.569 |
| Chronic renal failure | 3.28 (2.04–5.25) | <0.001 | 6.91 (4.30–11.1) | <0.001 | 0.028 |
| Severe LV dysfunction | 3.86 (2.52–5.89) | <0.001 | 6.89 (3.83–12.4) | <0.001 | 0.414 |
| Disease extent (3 vessel disease) | 1.98 (1.27–3.11) | 0.003 | 1.69 (1.13–2.53) | 0.011 | 0.523 |
| **CTO lesion-related revascularization** |  |  |  |  |  |
| CTO-PCI |  |  |  |  |  |
| CTO site (LCx) | 0.75 (0.42–1.34) | 0.328 | 0.72 (0.44–1.18) | 0.196 | 0.904 |

^*^For spontaneous MI and stroke, independent predictors of clinical outcomes in each treatment group could not be determined due to low outcome rates.

CI, confidence interval; CTO, chronic total occlusion; LCx, left circumflex coronary artery; LV, left ventricle; MI, myocardial infarction; NA, not available; PCI, percutaneous coronary intervention.

**Supplementary Figure 1**

**
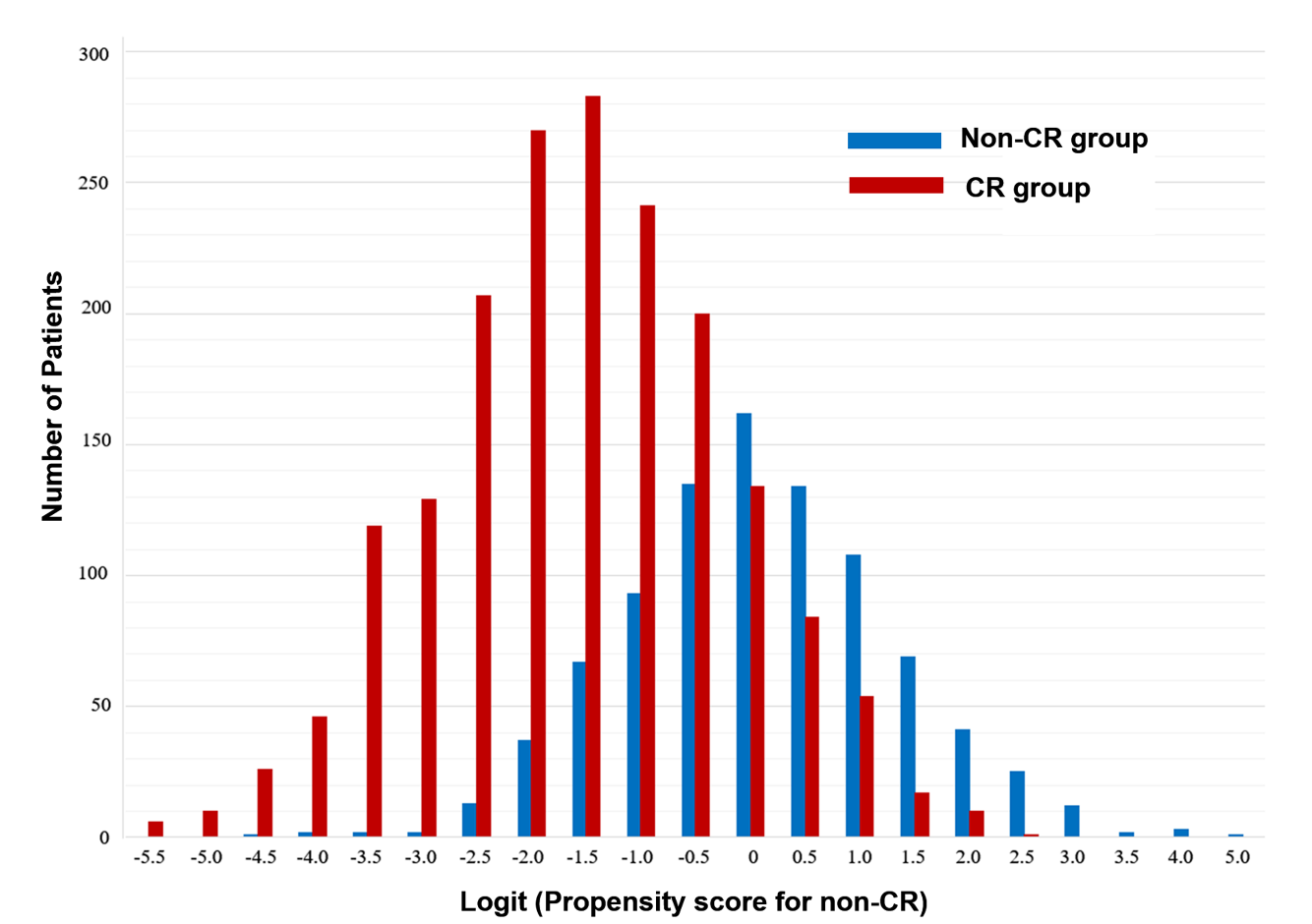
**

The propensity score for non-CR cases was defined as the probability that a patient from either the CR or non-CR group would be selected for deferred CTO-PCI. The logit of the propensity score ranged from –infinity to +infinity and was equal to the probability value for OMT one-on-one.

CR, complete revascularization; PCI, percutaneous coronary intervention.

**Supplementary Figure 2.**

**
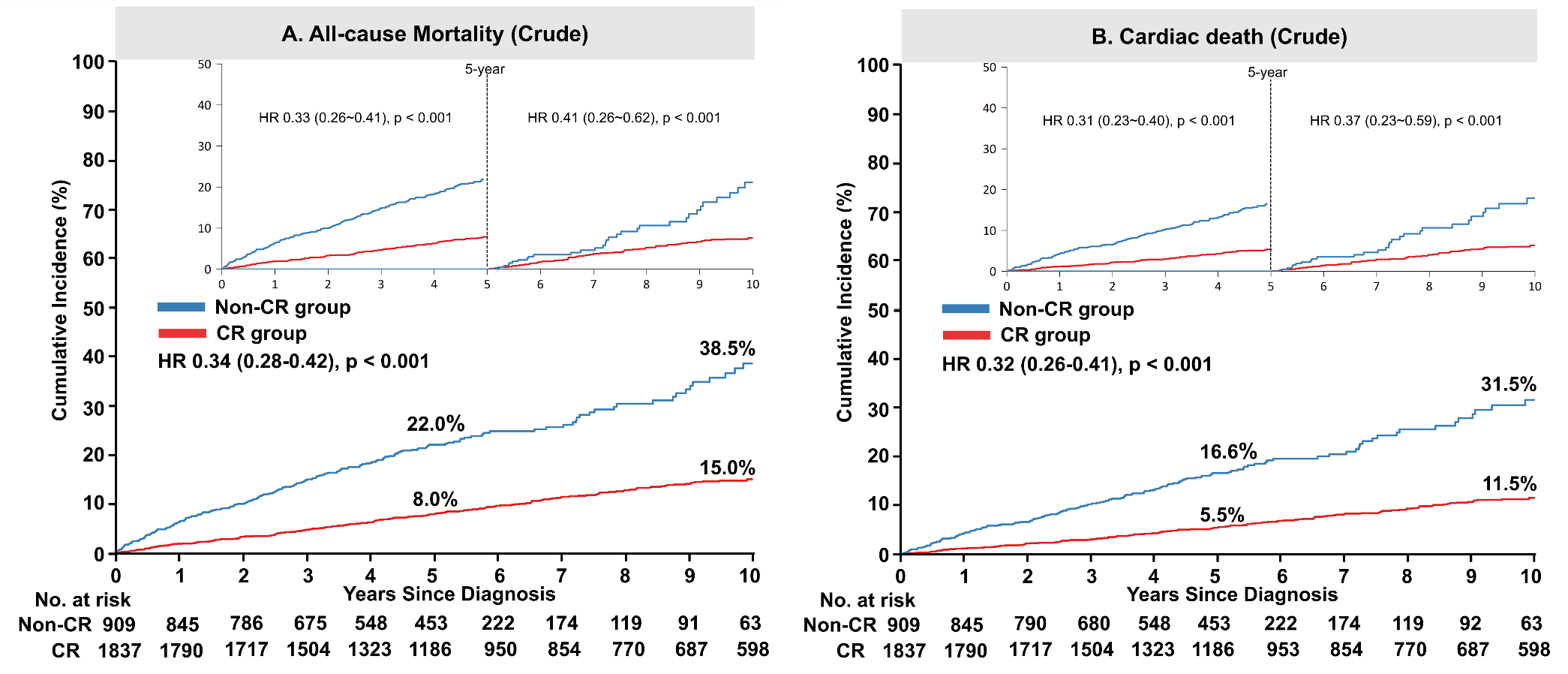
**

Risks of death in patients who did and did not achieve complete revascularization. Crude event curves for all-cause mortality (A) and cardiac death (B) in the non-CR and CR groups. HRs and 95% CIs are shown for the CR group versus the non-CR group.

CIs, confidence intervals; CR, complete revascularization; HRs, hazard ratios.

**Supplementary Figure 3.**

**
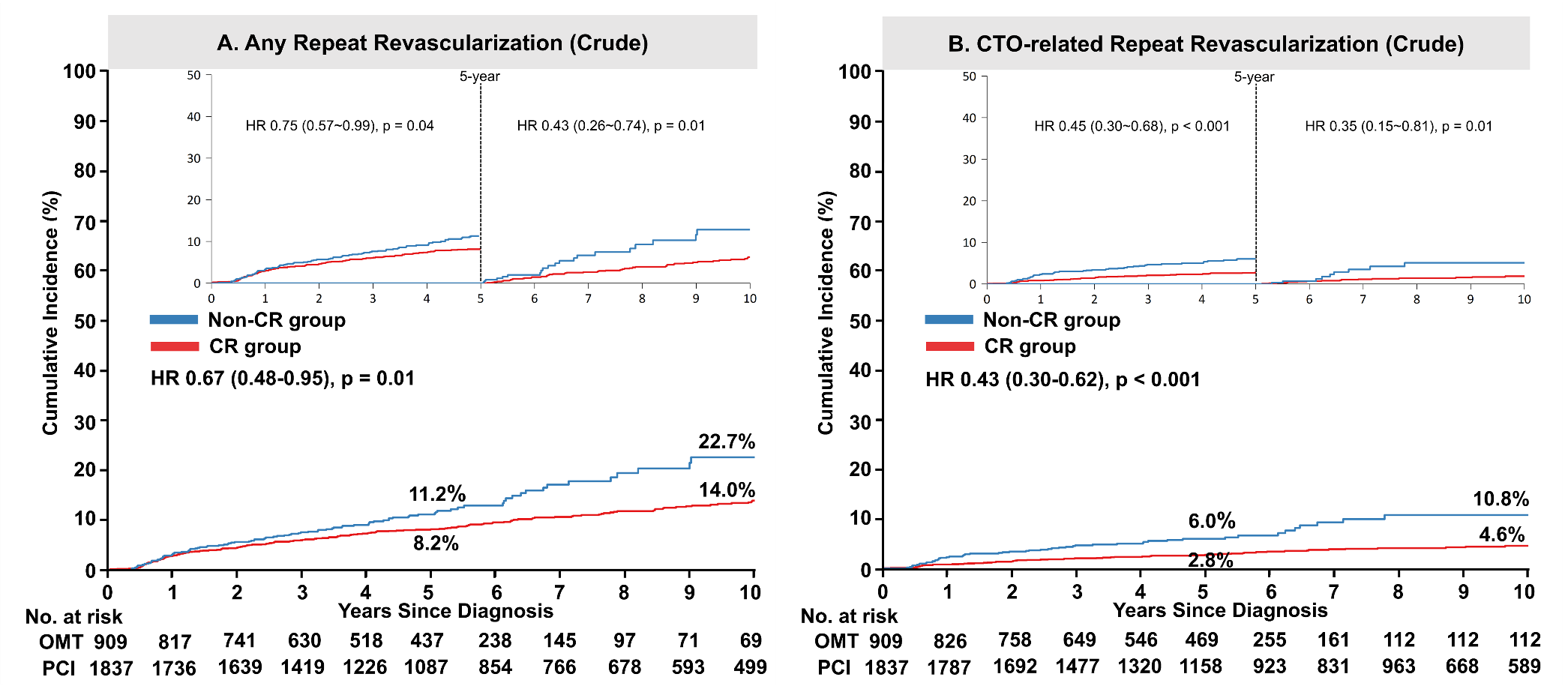
**

Risks of repeat revascularization in patients who did and did not achieve complete revascularization. Crude event curves for any repeat revascularization (A) and CTO-related repeat revascularization (B) in the non-CR and CR groups. HRs and 95% CIs are shown for the CR group versus the non-CR group.

CIs, confidence intervals; CR, complete revascularization; HRs, hazard ratios.
